# Geo-temporal patterns to design cost-effective interventions for zoonotic diseases -the case of brucellosis in the country of Georgia

**DOI:** 10.3389/fvets.2023.1270505

**Published:** 2023-12-20

**Authors:** Ariel L. Rivas, Stephen D. Smith, V. Basiladze, Tengiz Chaligava, Lile Malania, Irma Burjanadze, Tamar Chichinadze, Nikoloz Suknidze, Nana Bolashvili, Almira L. Hoogesteijn, Kendra Gilbertson, Jonathan H. Bertram, Jeanne Marie Fair, Colleen T. Webb, Paata Imnadze, Michael Kosoy

**Affiliations:** ^1^Center for Global Health, Internal Medicine, School of Medicine, University of New Mexico, Albuquerque, NM, United States; ^2^Geospatial Research Services, Ithaca, NY, United States; ^3^National Food Agency, Ministry of Environmental Protection and Agriculture of Georgia, Tbilisi, Georgia; ^4^National Center for Disease Control and Public Health, Tbilisi, Georgia; ^5^Vakhushti Bagrationi Institute of Geography, Ivane Javakhishvili Tbilisi State University, Tbilisi, Georgia; ^6^Department of Human Ecology, CINVESTAV, Merida, Yucatan, Mexico; ^7^Graduate Degree Program in Ecology, Department of Biology, Colorado State University, Fort Collins, CO, United States; ^8^Genomics and Bioanalytics, Los Alamos National Laboratory, Los Alamos, NM, United States; ^9^KB One Health LLC, Fort Collins, CO, United States

**Keywords:** brucellosis, zoonosis, geo-temporal analysis, Georgia (country), cost-benefit analysis

## Abstract

**Introduction:**

Control of zoonosis can benefit from geo-referenced procedures. Focusing on brucellosis, here the ability of two methods to distinguish disease dissemination patterns and promote cost-effective interventions was compared.

**Method:**

Geographical data on bovine, ovine and human brucellosis reported in the country of Georgia between 2014 and 2019 were investigated with (i) the Hot Spot (HS) analysis and (ii) a bio-geographical (BG) alternative.

**Results:**

More than one fourth of all sites reported cases affecting two or more species. While ruminant cases displayed different patterns over time, most human cases described similar geo-temporal features, which were associated with the route used by migrant shepherds. Other human cases showed heterogeneous patterns. The BG approach identified small areas with a case density twice as high as the HS method. The BG method also identified, in 2018, a 2.6–2.99 higher case density in zoonotic (human and non-human) sites than in non-zoonotic sites (which only reported cases affecting a single species) –a finding that, if corroborated, could support cost-effective policy-making.

**Discussion:**

Three dissemination hypotheses were supported by the data: (i) human cases induced by sheep-related contacts; (ii) human cases probably mediated by contaminated milk or meat; and (iii) cattle and sheep that infected one another. This proof-of-concept provided a preliminary validation for a method that may support cost-effective interventions oriented to control zoonoses. To expand these findings, additional studies on zoonosis-related decision-making are recommended.

## 1 Introduction

The COVID-19 pandemic brought many lessons and questions. One of them refers to whether epidemics should be countered with reactive or anticipatory approaches (1). To demonstrate why anticipatory approaches are necessary, cost–benefit oriented studies are needed.

*How, when* and *where* can implemented interventions lead to cost–benefit based results? To answer this composite question, the type of data analyzed is critical. The analysis of geo-referenced and temporal infectious disease-related data may determine whether intervening at specific geographical sites induce cost-effective policies (2).

Because numerous (if not infinite) geo-temporal patterns may be found in disseminating infectious diseases, geo-referenced and temporal data may also inform on covariates, such as soil, elevation, meteorology, seasonality, and sociology (3). Because they can –visually– reveal interactions, geo-referenced data can inform more than tabular data (4). Because the geographical context surrounding diseases may be unique and it may influence (promoting or preventing) their dissemination, geo-temporal analysis of diseases can capture relationships that reductionist approaches may omit or not anticipate (5, 6).

One specific question that decision-makers need to answer is where, exactly, interventions may lead to less costly, earlier and/or more beneficial results (7). To develop geo-referenced, decision-making oriented analyses, inter/transdisciplinary approaches have been recommended (8). Such approaches may consider bio-geographical and dynamical data that may feed models meant to interrupt disease transmission and/or be cost-effective (9).

While policy-making based on geo-referenced data has been promoted (10) and several studies have explored brucellosis (11–13), the overall as well as the specific (construct, internal, external and/or statistical) validity of such procedures have not yet been emphasized (14, 15). For example, external validity (which refers to multiple variables, metrics, locations, populations, and/or outcomes) has been reported to be infrequently evaluated with empirical data (16).

Geographically explicit, high-resolution, grid-based maps offer an actionable alternative to explore many sources of validity. Such maps have been used to investigate (non-infectious) interactions involving human and non-human species (17). These maps also circumvent the limitations of maps based on aggregate data, which miss local interactions among geo-referenced variables (18). In contrast, grid-based maps can display a high level of granularity (19). Furthermore, bio-geographical methods do not assume space homogeneity –an assumption associated with classic spatial statistics, which considers that neighbors are epidemiologically similar (20, 21).

Brucellosis-related dissemination patterns can be explored in Georgia, the country located in the South Caucasus. With brucellosis being a substantial endemic problem (22), Georgia has a large geo-temporal dataset on cases affecting cattle, sheep, and humans.

Such a context is also adequate to explore *One Health* processes, in which the environment interacts with potential hosts and non-human and human species may infect one another (23, 24). While numerous educational programs now focus on *One Health*, inter−/trans-disciplinary educational gaps have been reported in this field (25).

To evaluate cost–benefit oriented approaches, new concepts may be investigated. For example, the detection of small geographical sites where infections induced by the same bacterium affect two or more species may be desirable.

Accordingly, this study pursued two objectives: (i) to distinguish geo-temporal patterns of zoonotic disease dissemination, and (ii) to identify sites where interventions may induce cost-effective results.

## 2 Materials and methods

### 2.1 Data

Data on (ruminant) brucellosis were collected by the National Food Agency of the Ministry of Environmental Protection and Agriculture of Georgia between 2014 and 2019. Human data on brucellosis cases reported between 2015 and 2020 were provided by the Center for Disease Control and Public Health of Georgia. Because the purpose of this study was to generate a preliminary evaluation of a geo-referenced tool based on historical data, no inferences are here made on laboratory-related issues (such as the bacteriological tests used) or epidemiological-related issues (such as estimates of disease prevalence).

The data were filtered to extract geographically referenced and time-stamped records (records with latitude, longitude, and date) for cattle, sheep, and humans. This filtering resulted in the identification of 7,643 records for the 2014–2019 period (2,999 cattle and 4,644 sheep). Nine hundred and ninety-three human records were identified between 2015 and 2018. Due to space limitations of Brief Reports, human data from 2014 and 2019 are not reported but are available upon request.

### 2.2 Geo-referenced method

These tabular data were brought into a geographic information system (*ArcGIS Pro 3.1.3*, ESRI, Redlands, CA, USA) and a geodatabase point feature was generated. Starting with this initial point feature information, yearly species-specific point features were generated for mapping purposes.

To identify areas of case concentrations, a 5 km-by-5 km, country-wide grid (7,068 cells) was created, and scripts were developed to associate case data with the enclosing grid cell. Such size was selected as a compromise between larger areas (more likely to capture more cases but less precise in terms of specific case geo-location) and smaller areas (more precise in terms of specific case geo-location but more likely to exclude nearby cases).

Each yearly species-specific grid cell polygon feature was added to an *ArcGIS Pro* map and summarized by grid cell number. Each summarized table was exported to a text file for tabulation and analysis. Using the summarized data, a quantile (maximum of three) display of the grid cell’s total cases was generated in *ArcGIS Pro*, and a map layout was created.

### 2.3 Construct, internal, external and statistical validity

The degree to which the concept of interest was actually investigated by the operation implemented (construct validity) was estimated by comparing the bio-geographical (BG) model with an alternative –the Getis-Ord Gi* or Hot Spot (HS) analysis, a method performed by *ArcGIS Pro* that assumes neighbors are bio-geographically similar. The method that yielded the highest case density (cases/square kilometer) was viewed as the most cost-effective.

Internal validity (lack of confounding) was assessed by testing several variables. Threats to internal validity were ruled out when two or more variables yielded similar results.

Statistical analysis was performed using *Minitab 22* (Minitab Inc., State College, PA, USA). Regression analysis explored relationships between the number of cases reported at sites where ruminants, humans, or both human and non-human species reported infections. By investigating two or more host species over two or more years, the external validity of the BG method was also explored.

## 3 Results

**Figure 1:**
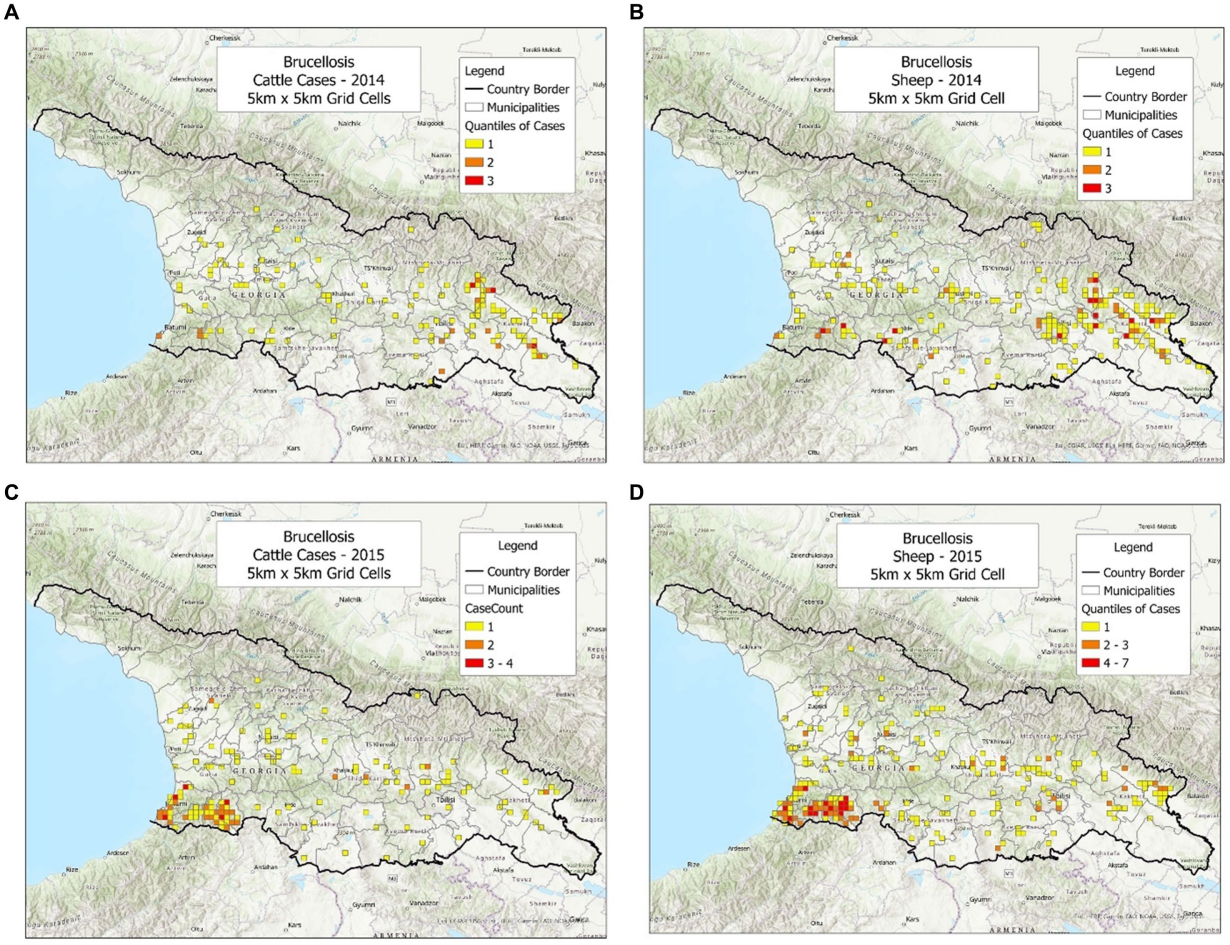
Brucellosis cases reported in cattle and sheep, in 2014–2015. **(A)** Cattle cases, 2014. **(B)** Sheep cases, 2014. **(C)** Cattle cases, 2015. **(D)** Sheep cases, 2015.

**Figure 2:**
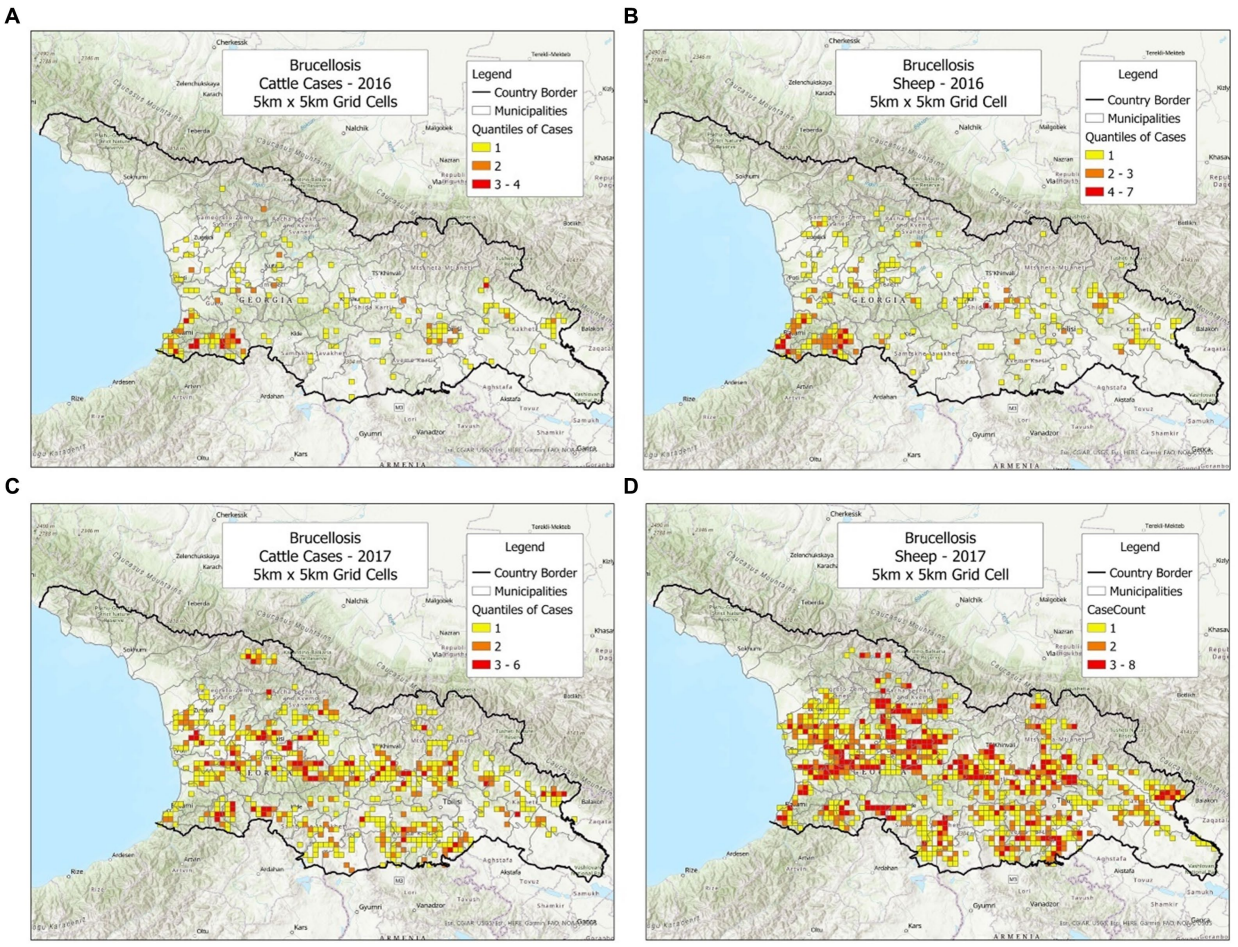
Brucellosis cases reported in cattle and sheep, in 2016–2017. **(A)** Cattle cases, 2016. **(B)** Sheep cases, 2016. **(C)** Cattle cases, 2017. **(D)** Sheep cases, 2017.

**Figure 3:**
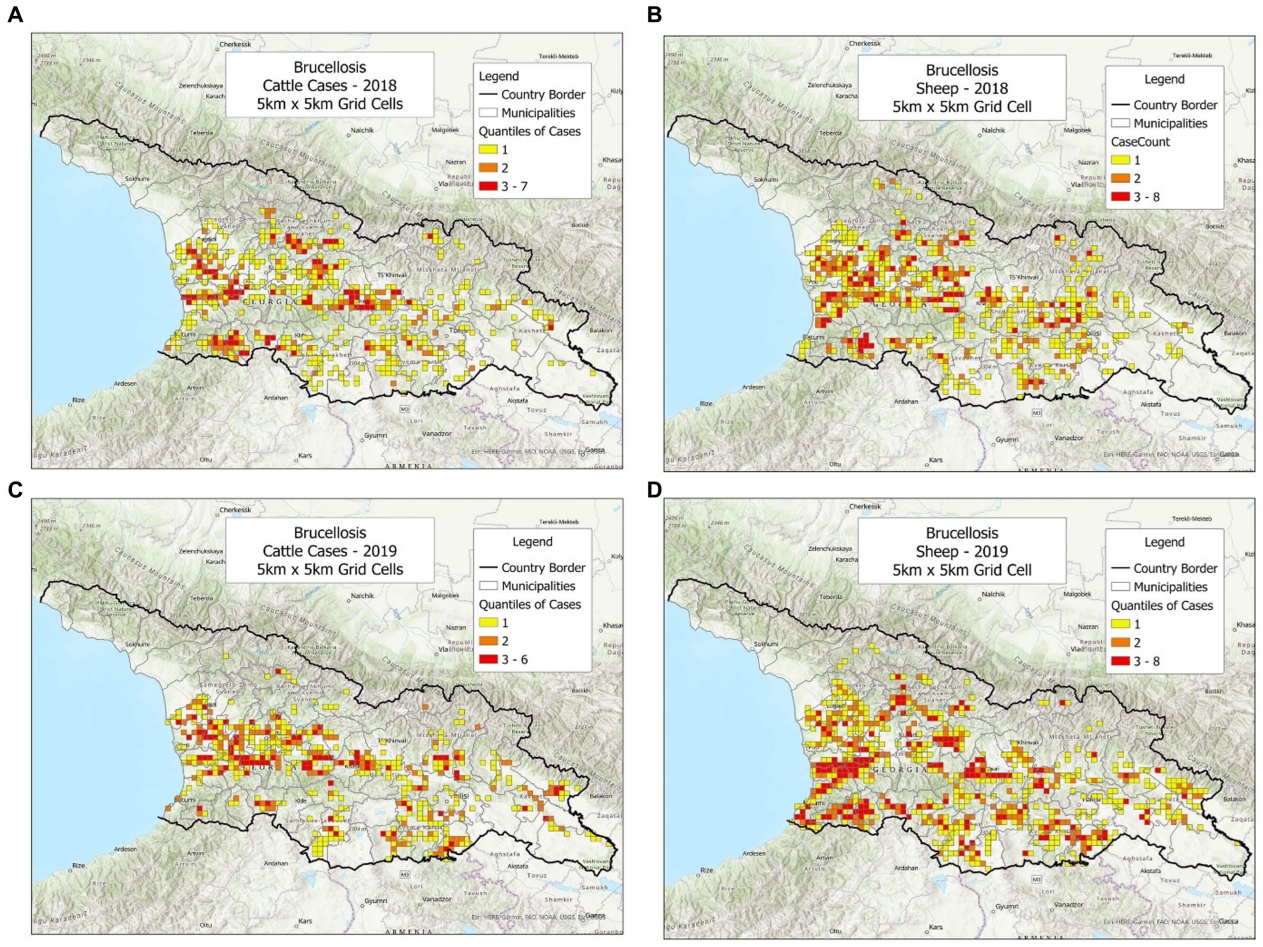
Brucellosis cases reported in cattle and sheep, in 2018–2019. **(A)** Cattle cases, 2018. **(B)** Sheep cases, 2018. **(C)** Cattle cases, 2019. **(D)** Sheep cases, 2019.

To investigate whether the bio-geographical tool could be used in different populations and/or different timeframes, cattle and sheep cases were plotted, side by side, annually (Figures 1–3). It was observed that cattle cases (Figures 1A, 2A, 3A) matched sheep cases (Figures 1B, 2B, 3C). However, time did not appear to be related with case location. For instance, earlier cases (Figures 1A,B) did not match later cases, even when a short temporal period (a year) was considered (Figures 2A,B).

**Figure 4:**
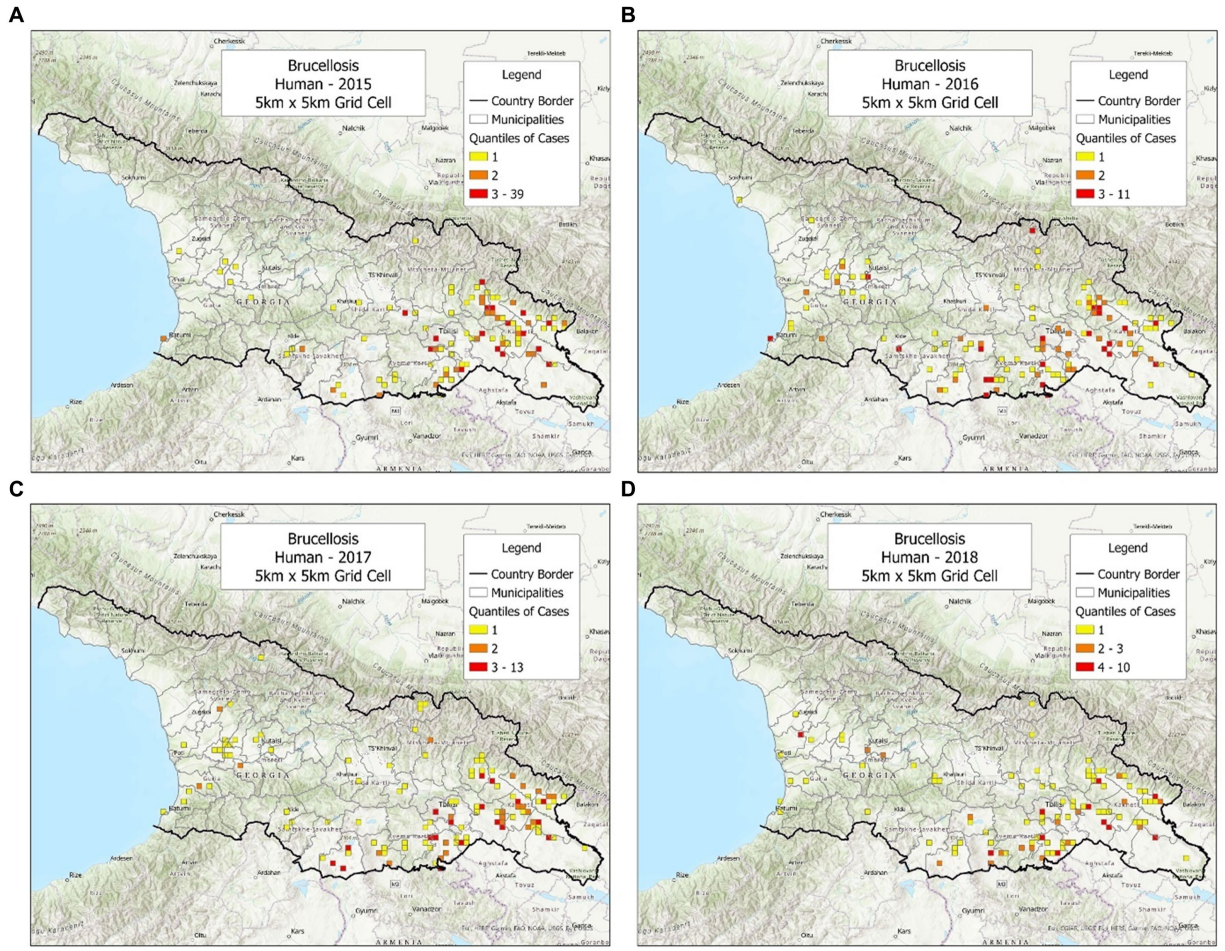
Brucellosis cases reported in humans, in 2015–2018. **(A)** Cases reported in 2015. **(B)** Cases reported in 2016. **(C)** Cases reported in 2017. **(D)** Cases reported in 2018.

While cattle and sheep cases displayed noticeable geo-referenced changes over time, most human cases did not. Over 4 years, most human cases were reported in the same area –the eastern region of Georgia (Figures 4A–D). Yet, a second pattern associated with human cases was also seen, which was heterogeneous and took place in the central municipalities of Georgia (Figures 4A–D).

Sites that reported cases affecting two species (cattle and sheep or ruminant sites) seemed to differ from the remaining sites. When the counts or percentages of cases and sites were considered, ruminant sites exhibited twice as many cases as uni-species sites (Supplementary Table 1).

Findings supported the differentiation of brucellosis cases into three geo-temporal patterns: (i) one only observed in the eastern region, which included human cases; (ii) one also affecting humans, which took place outside the eastern region; and (iii) non-human cases, reported outside the eastern region. Within the last variety, two presentations were distinguished: (a) sites where only one ruminant species was infected, and (b) sites where both cattle and sheep cases were reported (ruminant sites).

Findings also suggested an additional pattern of cattle and sheep cases, which was observed in the south-western region of the country, in 2015. Upon further investigation, it was found that the geographical location did not characterize cattle/sheep farms and/or summer pastures but the location of specialized laboratories (located close to ports) where animals exported to other countries are tested before they are embarked. This unexpected finding illustrates the usefulness of geo-referenced data analysis, which may shed light on procedures recorded but not always considered in routine epidemiological analyses.

While the historical nature of the data prevented inferences and predictions, possible educational applications included data-driven hypotheses on peak temporal patterns reaching in 2017 (Supplementary Figure 1). The number of cases observed in either ruminant species predicted the total number of cases found in multi-species (ruminant) sites (*p* < 0.01, Supplementary Figure 2). In one scenario under study, the bio-geographical procedure captured more cases than the HS analysis (583 vs. 521, respectively) in an area 28.4% smaller than the area occupied by the HS solution (Supplementary Figures 3A,B). Because each unit of area occupied 25 square kilometers, expressed as case density/square kilometer, the BG method captured 0.167 cases/sq km while the HS analysis detected 0.107 cases/sq km. Consequently, the case density of the BG approach was 56.2% higher than that of the HS. This difference in potential cost-effectiveness was explained by two factors: (i) the HS missed large areas that included numerous cases (Supplementary Figure 3C) and, (ii) in particular, the HS analysis did not detect nine mini-areas with very high case density (Supplementary Figure 3D). If cost effectiveness of interventions was measured as the ratio of benefits over costs (cases captured/area unit), then the ratio of the BG method would be 2.18 (156.2/71.6), i.e., the BG method exhibited a benefit/cost ratio twice as large as the one shown by the HS analysis.

To explore possible applications in decision-making, the BG method was further investigated according to the (zoonotic vs. non-zoonotic) content of the infected sites. Using the data reported in 2018, the case density of sites reporting zoonotic (human, cattle and/or sheep) cases was at least 2.6 and up to three times higher than the number of sites where only one species was infected (Supplementary Figure 4). Accordingly, if a policy meant to be cost-effective was designed to be applied in this scenario, the first priority of interventions would focus on zoonotic sites (locations that reported a high case density where humans and non-humans were infected), non-zoonotic sites (where only one –human or non-human– species was infected) would be the second priority and, as the last priority, non-zoonotic sites reporting the lowest case density would be intervened.

## 4 Discussion

### 4.1 Caveats

Findings are prone to bias due to many factors, which include but are not limited to the varying frequency and magnitude of testing conducted over time and the threats to validity associated with the analysis of historical data (26). Accordingly, this study should not be construed to represent the current status of brucellosis-related conditions existing in Georgia but, instead, a realistic learning scenario that can support research and education in geo-epidemiology.

While zoonotic infections disseminate by only three (direct, indirect, or both direct and indirect) types of transmission, the expression of such transmissions may vary according to the local geography. Consequently, cost-effective interventions may be designed and selected according to specific geo-temporal expressions.

### 4.2 Geo-geographical patterns of zoonotic disease transmission

The hypothesis of direct contacts between non-human infected species and susceptible humans was supported by the pattern observed in the eastern region of Georgia (27). Disease transmission, in this modality, is thought to be facilitated when migrant shepherds move their sheep, twice a year, between the southern and the northern borders of the country, along a path flanked by mountains –a geographical feature that determines a rather constant geographical pattern, detected regardless of time (Figures 4A–D). In contrast, other human infections (reported outside the eastern region) are possibly facilitated by the consumption of contaminated milk and meat (26). The hypothesis of cattle-sheep contacts (apparent in several regions of Georgia) is facilitated by a common agricultural practice –also observed in many countries–, in which cattle and sheep share summer pastures (28).

This study emphasized high-resolution, geo-referenced and cost-effective epidemic control measures. To avoid loss of resolution, this study was not centered on municipalities (which differ in area, population, connectivity and many other aspects) but on small areas of equal size (cells of 25 sq km). Such operation facilitated the detection of specific geographical sites where infections affecting two or more host species were found. While approaches that aggregate data and assume homogeneous data distributions over large spatial areas tend to result in large areas to be intervened (e.g., higher costs), the geo-biological method identified small areas with high case density which, if intervened, are likely to induce more beneficial interventions (more cases to be covered) at lower costs (in smaller areas to be intervened), and –due to their smaller areas– such interventions may be completed earlier. Furthermore, small areas with high case density can be mapped over other layers and, consequently, inform on connectivity and many other variables, as described before (29, 30).

Such a geographical approach can be complemented with a biological emphasis.

When the data are divided into classes (e.g., zoonotic or human and non-human, only ruminants, and only humans), the number of cases may be much higher in zoonotic sites and, consequently, interventions that prioritize such sites may be less costly and/or more beneficial (Supplementary Figure 4).

### 4.3 Validation

Several estimates of construct, internal, external and/or statistical validity were facilitated by the method under study. Although the historical nature of the data is prone to several threats to validity, the fact that three host species were investigated (and tested for several years) demonstrates that this tool may possess external validity. Because the analysis of five variables yielded similar patterns (and, therefore, confounding was ruled out) and revealed statistically significant associations, internal and statistical validities were supported (Supplementary Figure 1). Because the bio-geographical approach captured more cases per sq. km than the alternative method –a finding also observed when geographical locations reporting infections were classified according to their ability to generate or disseminate zoonotic cases–, construct validity was not ruled out (Supplementary Figures 3, 4).

However, the previous comparison did not consider all possible scenarios (which may include additional bio-geo-temporal variables). To better estimate cost-effective interventions, the benefits associated with interrupting disease transmission (especially, long-term interactions) need to be considered (9).

### 4.4 Potential applications and future studies

These geo-referenced findings facilitated the development of, at least, five new studies or projects. One refers to prioritizing interventions in locations that report human and non-human cases (‘zoonotic disease’ sites).

Because such sites can induce secondary infections along ruminant and non-ruminant hosts, they may be prioritized when cost–benefit oriented interventions are planned. Support for ranks that prioritize where interventions should be implemented may be facilitated by a cost–benefit oriented analysis. For example, when there is evidence that zoonotic mini-sites capture more cases than sites presenting infections in a single species (as shown in Supplementary Table 1; Supplementary Figure 4), zoonotic sites could be prioritized, followed by sites that do not show zoonotic cases, and, finally, sites that display the lowest case density and are not zoonotic. Such priority ranks could later be modified or expanded when additional information becomes available. For example, earlier cases could receive the first priority to be intervened when they also display the shortest inter-case distance along actual road networks (30).

The second potential intervention refers to migrant shepherds. Because their cases are consistently reported, every year, at the same places, it appears that some behaviors are repeated over time, which occur at specific places where interventions are likely to be beneficial. Because available information suggests there may be inadequate brucellosis-related, educational campaigns in the Tusheti and Kakheti regions (22), future investigations may focus on sociological-educational variables at specific geographical areas, such as associations between educational packages received on the role of uncooked meat and/or unpasteurized milk consumption and disease occurrence (31).

Third, findings may support new studies that seek to identify covariates associated with zoonotic sites. To that end, inverse-problem methods could be considered. Such approaches start with a known solution and then attempt to identify the predictors (32). Inverse-problem approaches have been proposed for epidemiological studies (33). In this case, the precise geo-referenced location of ‘zoonotic sites’ (a site that may be associated with a rather unique combination of factors) could be the starting point for such inquiries.

Furthermore, future studies based on these or similar geo-referenced procedures could explore alternative methods that may estimate the costs induced by bacterial zoonoses and the potential benefits of preventive campaigns. While similar studies have been conducted for viral zoonoses (1), no cost–benefit estimates on bacterial zoonoses-related decision-making have been emphasized. One exception in this area refers to Mongolia, where a 3.2 benefit/cost ratio has been reported (34). Future studies on cost effectiveness may also consider pastoralist practices (35).

Last, but not least, the development of new educational programs is recommended. It is suggested that, to generate and validate new tools that integrate three fields (epidemiology, geographical data analysis and decision-making), the academic creation of such an interdisciplinary field is required.

## 5 Conclusion

It is suggested that the geo-temporal analysis of brucellosis may be instrumental to investigate and teach how zoonoses emerge and disseminate. Methods similar to the one reported may be considered in the design of geographical site-specific, time-sensitive, cost-effective interventions.

## Data availability statement

The original contributions presented in the study are included in the article/Supplementary material, further inquiries can be directed to the corresponding author.

## Author contributions

AR: Conceptualization, Writing – original draft. SS: Formal analysis, Visualization, Writing – review & editing. VB: Data curation, Supervision, Writing – review & editing. TCha: Data curation, Writing – review & editing. LM: Data curation, Project administration, Writing – review & editing. IB: Data curation, Writing – review & editing. TChi: Data curation, Writing – review & editing. NS: Data curation, Writing – review & editing. NB: Data curation, Supervision, Writing – review & editing. AH: Investigation, Writing – review & editing. KG: Data curation, Writing – review & editing. JB: Data curation, Writing – original draft. JF: Writing – review & editing. CW: Project administration, Writing – review & editing. PI: Supervision, Writing – review & editing. MK: Conceptualization, Funding acquisition, Writing – original draft.
