# Supplementary material for: Geo-temporal patterns to design cost-effective interventions for zoonotic diseases -the case of brucellosis in the country of Georgia
Source: Front Vet Sci. 2023 Dec 20;10:1270505. doi: 10.3389/fvets.2023.1270505 (PMC10765567; doi:10.3389/fvets.2023.1270505)

Supplementary Figure 1. Estimates of internal and external validity. Five variables (including two metrics - counts or percentage- and two species -cattle and sheep populations-) supported the hypothesis that, within the timeframe investigated, brucellosis peaked in 2017 (blue rectangle).

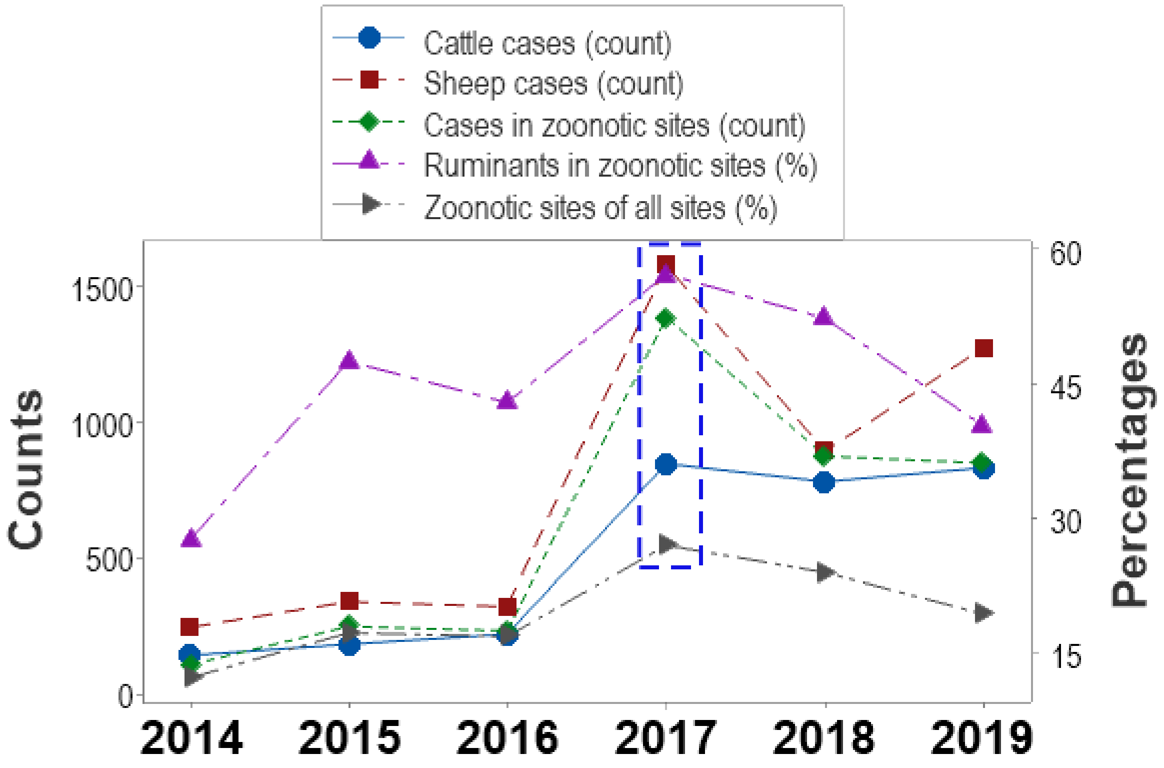

Supplementary Figure 2. Estimates of external and statistical validity. Data from two ruminant species conveyed similar inferences: there is a linear and statistically significant relationship ($p<0.01$) between case counts of (either species) and the case count of ruminant sites. **A**: Cattle data. **B**: Sheep data.

A

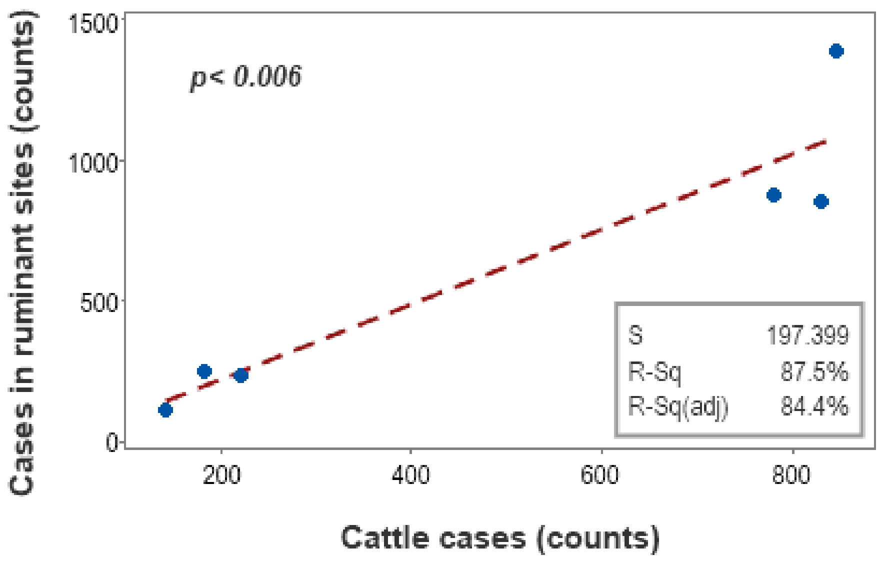

B

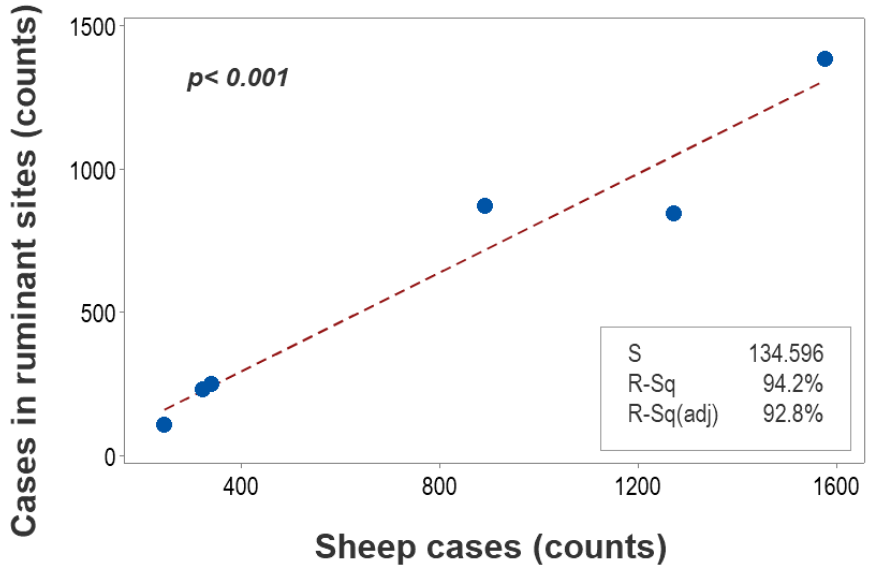

Supplementary figure 3. Evaluation of construct validity.
To assess cost-effectiveness, the same data were analyzed by the bio-geographical method (BG) and the Hot Spot analysis (HS). Using ruminant data from 2018 as an example, it is shown that the BG method identified139 25-sq km orange and red squares, which included 583 cases or 4.19 cases/square (583/139, **A**). In contrast, the HS analysis found 521 cases in 194 squares or 2.68 cases/square (521/194, **B**). Consequently, the case density of the BG approach was 56.3% higher (4.19/2.68) than that of the HS. This difference in potential cost-effectiveness was explained by two factors: (i) the HS missed large areas that included numerous cases (**C**) and, (ii) in particular, the HS analysis missed nine mini-areas with a very high case density (**D**). Such a difference was achieved while the BG analysis occupied an area 28.4% smaller (139/194 squares or 71.6%) than the area covered by the HS analysis. If cost effectiveness of interventions was measured as the ratio of benefits over costs (here expressed as cases captured/area unit), then the ratio of the BG method would be **2.18** (156.3/71.6), i.e., the BG method exhibited a benefit/cost ratio twice as large as the one shown by the HS analysis.

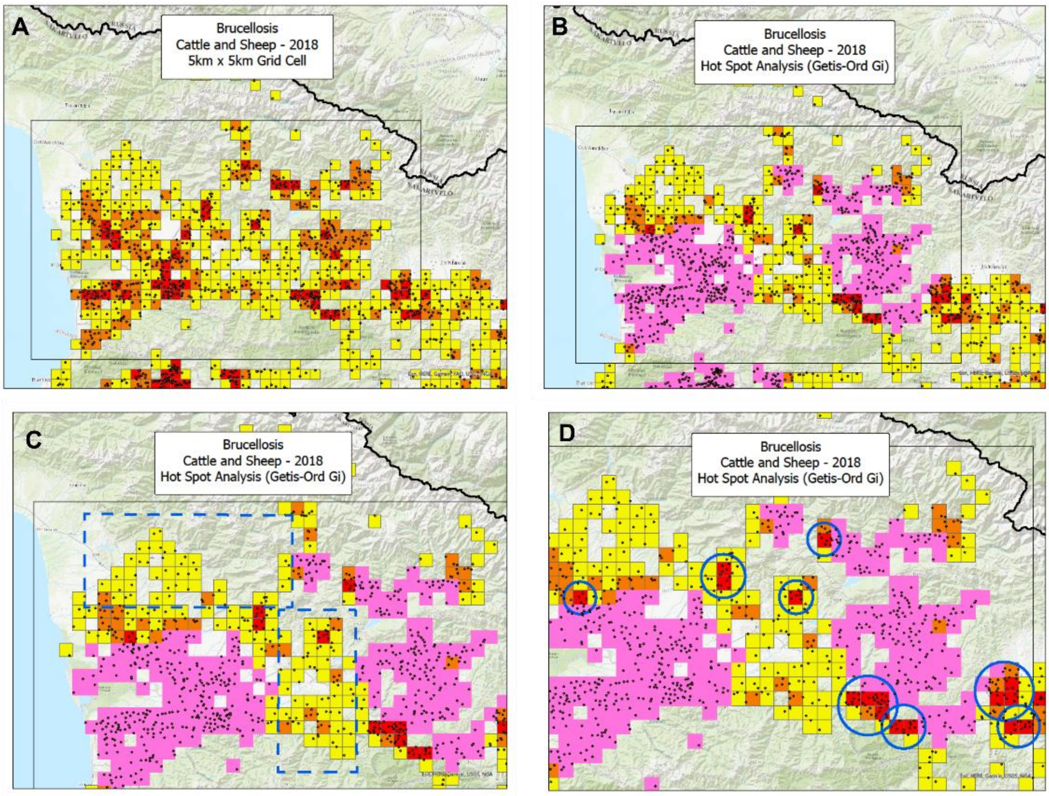

<u>Supplementary figure 4. A bio-geographical analysis may guide cost-benefit oriented, prioritized interventions.</u> Using the BG method to explore the contribution of site types to the 2018 data, it is shown that zoonotic sites (geographical locations where human and non-human cases were detected) displayed a case density between 2.6 and 2.99 times as high as any site that only included non-zoonotic cases (i.e., those that only reported human, cattle, or sheep cases). This metric –together with additional geo-bio-temporal information related to such sites– may provide decision-makers with a guide on where interventions may be prioritized. In this scenario, sites that report a high density of zoonotic cases could be the first priority, followed by sites where non-zoonotic cases are reported with a high case density, and finally, sites that report a low case density involving any species.

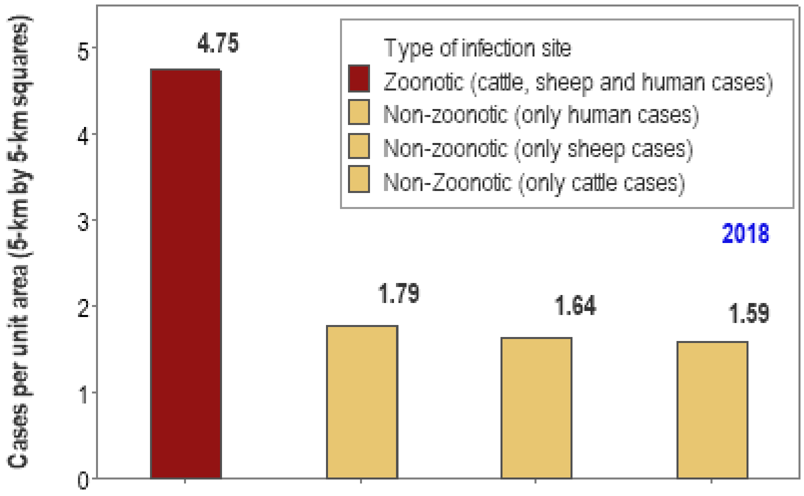

Supplement: Supplementary file 1 [file Data_Sheet_1.pdf]
