# Supplementary material for: Geo-temporal patterns to design cost-effective interventions for zoonotic diseases -the case of brucellosis in the country of Georgia
Source: Front Vet Sci. 2023 Dec 20;10:1270505. doi: 10.3389/fvets.2023.1270505 (PMC10765567; doi:10.3389/fvets.2023.1270505)
Supplement: Supplementary file 2 [file Table_1.DOCX]

**Supplementary Table 1. Brucellosis cases reported in ruminants between 2014 and 2019.**

| Year | **2014** | **2015** | **2016** | **2017** | **2018** | **2019** |
| --- | --- | --- | --- | --- | --- | --- |
| Cattle cases (count) | 142 | 183 | 220 | 845 | 779 | 830 |
| Sheep cases (count) | 245 | 339 | 321 | 1576 | 891 | 1272 |
| Cattle & sheep cases (sum of counts) | 387 | 522 | 541 | 2421 | 1670 | 2102 |
| Cases (count) found in sites with both cattle and sheep (ruminant) cases | 107 | 248 | 232 | 1384 | 873 | 847 |
| Percentage of all cattle  and sheep cases found in ruminant sites | 27.6 % (107/387) | 47.5 % (248/522) | 42.9 % (232/541) | 57.2 %  (1384/ 2421) | 52.3 % (873/1670) | 40.3 % (847/2102) |
| Sites with cattle cases (count) | 122 | 144 | 170 | 558 | 491 | 484 |
| Sites with sheep cases (count) | 196 | 214 | 221 | 829 | 552 | 674 |
| Number of cattle and  sheep sites (sum of counts) | 318 | 358 | 391 | 1387 | 1043 | 1158 |
| Sites with both cattle and sheep cases (count) | 39 | 62 | 66 | 374 | 250 | 225 |
| Percentage of ruminant sites out of all sites | 12.3%  (39/318) | 17.3 % (62/358) | 16.9% (66/391) | 27.0 %  (374/1387) | 24.0 % (250/1043) | 19.4 % (225/1158) |
| Average number of  cases in cattle sites | 1.16 (142/122) | 1.27 (183/144) | 1.29 (220/170) | 1.51  (845/558) | 1.59 (779/491) | 1.71 (830/484) |
| Average number of  cases in sheep sites | 1.25  (245/196) | 1.58 (339/214) | 1.45 (321/221) | 1.90 (1576/829) | 1.61 (891/552) | 1.89 (1272/674) |
| Average number of cases in ruminant sites | 2.74  (107/39) | 8.41  (522/62) | 8.20  (541/66) | 6.47  (2421/374) | 6.68  (1670/250) | 9.34  (2102/225) |

Across time, between 27 and 57% of all ruminant cases were found in ruminant sites. Across time, the average number of cases was at least twice as large in ruminant sites than in sites where only one ruminant species reported infected cases. Specifically, that number was: (i) 2.2-2.4 times larger in 2014 (2.74/1.16, 2.74/1.25); (ii) 5.3-6.6 times larger in 2015 (8.41/1.27, 8.41/1.58); (iii) 5.7-6.4 times larger in 2016 (8.2/1.29, 8.2/1.45); 3.4-4.1 larger in 2017 (6.47/1.51; 6.47/1.9); (iv) 4.1 times larger in 2018 (6.68/1.59, 6.68/1.61); and (v) 4.9-5.5 times larger in 2019 (9.34/1.89, 9.34/1.71).
